# Which Codon Synonym Is Best? It May Depend on What's on the Menu

**DOI:** 10.1371/journal.pbio.1002014

**Published:** 2014-12-09

**Authors:** Richard Robinson

**Affiliations:** Freelance Science Writer, Sherborn, Massachusetts, United States of America

Every student of biology knows that the genetic code, which translates messenger RNA sequence into amino acid sequence, contains multiple synonyms, with the same amino acid specified by codon triplets that differ in the third, or “wobble,” position. AAU and AAC, for instance, both encode asparagine. However, while they specify identical amino acids, the two synonyms are not precisely the same, at least when it comes to the act of translation. Mechanistic studies show that there are subtle but significant differences in how each interacts with its corresponding transfer RNA (tRNA), differences that affect both the speed and the accuracy of translation. Thus, selection can, and does, act on the choice of synonym, leading to both cross species variation in synonyms for a particular amino acid position in homologous proteins and within-species variation in synonym frequency in genes expressed in different developmental stages.

Determining which codon synonyms provide the best combination of speed and accuracy is not easy, and those who study codon usage have largely assumed that there is a single “optimal” choice for each amino acid. However, in a new study in this issue of *PLOS Biology*, John Zaborske, Allan Drummond, and colleagues show that both across fruit fly species and within the fly developmental process, which codon is optimal depends, surprisingly, on the proportion of corresponding tRNAs that bear an unusual nucleotide, which, still more surprisingly, is likely a consequence of the availability of that nucleotide's precursor from bacterial food sources.

The unusual nucleotide is queuosine (Q), structurally similar to guanosine (G) and able to take its place in the triplet anticodon that sits in the wobble-binding position of the asparagine tRNA. Guanosine can be enzymatically converted to Q by combining it with queuine, derived from bacteria either from food or the gut biota.

The function of this modification is unknown, but it is clear that Q and G differ in their interaction with codons. The authors explored the implications of those differences in a kinetic model combining the effects of translation speed and accuracy. Specifically, based on the higher affinity for the ribosome of a Q-containing tRNA (Q-tRNA) versus a G-containing tRNA (G-tRNA), their model assumed that Q-tRNA can translate both asparagine synonyms faster than G-tRNA can. Furthermore, again based on measurements of differential binding affinities, they assumed that Q-tRNA can translate AAC much faster than it can translate AAU, while G-tRNA can translate AAC only slightly faster than it can translate AAU.

What about accuracy? Mistakes occur when the “wrong” tRNA binds to the target codon. For asparagine, one wrong tRNA is one that incorporates a threonine into the growing polypeptide chain and includes an inosine nucleotide (I-tRNA) in its wobble-binding spot. Again, nucleotide-based differences proved essential to predicting the effects of Q on accuracy.

Translation of a codon can be considered a competition between two teams (the “right” team and the “wrong” team). Speed is reflected in the total score of the game, and accuracy in the margin of victory, or the difference in scores.

In the translation of a U-ending codon, G-tRNA is a weak competitor, but I-tRNA (as a representative member of the wrong team) is much weaker. At the end of the game, the score is low, but the margin of victory is high. When a C-ending codon appears, the competition is much stiffer, with both right and wrong teams working faster. The final score is higher, but G-tRNA's margin of victory is lower, as the wrong team scores relatively more often.

**Figure 1:**
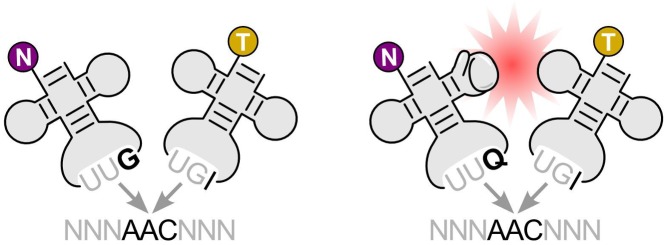
Modification of a tRNA with Q improves its ability to compete with other tRNAs during translation, altering codon accuracy and usage across the genome.

Q modification changes the game dramatically. Because it is so much faster than the competition when it comes to translating C-ending codons, Q-tRNA can now completely trounce the competition, yielding a wide margin of victory. Thus, in the presence of Q, translation of C-ending codons is both very fast and very accurate, which is enough to tip the balance away from an advantage for G-tRNA. In other words, Q-tRNA is the best player, and when Q is available, translating C-ending codons is the best game to play. In contrast, without Q, Q-tRNA cannot take the field, and translating U-ending codons is a better game (Figure 1).

To determine if the results predicted from the model applied across the actual fly genome, the authors quantified Q-tRNA in the fly as a whole at various stages of development and looked to see if its level correlated with peaks of expression of genes whose synonyms at highly conserved amino acids favored C's rather than U's. As predicted, they found that in the embryonic stage, elevation of Q-tRNA was high, as was expression of genes with a preference for C-ending synonyms. U-ending codons were favored in the larval and pupal stages, along with low levels of Q-tRNA. In the adult, the embryonic pattern reemerged. Similar results were seen across multiple species across the fruit fly genus.

The explanation for this developmental pattern may lie in the rapid cell division in the larva and pupal stages, the authors suggest. Without ever-increasing sources of queuine, the larva may not be able to supply enough of the precursor to make high levels of Q-tRNA. In that case, genes that must be translated at high levels during these stages will experience selective pressure for inclusion of the U-ending synonym, with its lower error rate in translation.

The detailed mechanistic explanation provided by the authors will need to be tested and refined in other systems before it becomes the last word on selection of synonyms in the genome. However, it seems clear that, as with spoken language, one synonym does not fit every situation, and as the context changes, the slight differences between them can be deeply important.

**Zaborske JM, Bauer DuMont VL, Wallace EWJ, Pan T, Aquadro CF, et al. (2014) A Nutrient-Driven tRNA Modification Alters Translational Fidelity and Genome-Wide Protein Coding across an Animal Genus.** doi:10.1371/journal.pbio.1002015

